# A Retrospective Analysis: Investigating Factors Linked to High Lung-RADS Scores in a Nonsmoking, Non-Family History Population

**DOI:** 10.3390/diagnostics14080784

**Published:** 2024-04-09

**Authors:** Chi-Shen Chen, Hsien-Chung Yu, Chun-Hao Yin, Jin-Shuen Chen, Yao-Shen Chen, I-Shu Chen

**Affiliations:** 1 Health Management Center, Kaohsiung Veterans General Hospital, Kaohsiung 813414, Taiwan or chirise@vghks.gov.tw (C.-S.C.); hcyu@vghks.gov.tw (H.-C.Y.); 2 Department of Nursing, Mei-ho University, Pingtung 91202, Taiwan; 3 Division of Gastroenterology and Hepatology, Department of Internal Medicine, Kaohsiung Veterans General Hospital, Kaohsiung 813414, Taiwan; 4 Department of Medical Education and Research, Kaohsiung Veterans General Hospital, Kaohsiung 813414, Taiwan; chyin@vghks.gov.tw; 5 Institute of Health Care Management, National Sun Yat-sen University, Kaohsiung 80421, Taiwan; 6 Department of Administration, Kaohsiung Veterans General Hospital, Kaohsiung 813414, Taiwan; dgschen@vghks.gov.tw (J.-S.C.); yschen@vghks.gov.tw (Y.-S.C.); 7 Division of General Surgery, Department of Surgery, Kaohsiung Veterans General Hospital, Kaohsiung 813414, Taiwan; 8 Department of Physical Therapy, Shu-Zen Junior College of Medicine and Management, Kaohsiung 82144, Taiwan

**Keywords:** lung cancer, LDCT screening, risk factors, high-risk group

## Abstract

Low-dose computed tomography screening for lung cancer is currently targeted at heavy smokers or those with a family history of lung cancer. This study aimed to identify risk factors for lung cancer in individuals who do not meet the current lung cancer screening criteria as stipulated by the Taiwan Health Promotion Agency’s low-dose computed tomography (LDCT) screening policy. A cohort analysis was conducted on 12,542 asymptomatic healthy subjects aged 20–80 years old who voluntarily underwent LDCT scans from January 2016 to December 2021. Logistic regression demonstrated that several factors, including age over 55 years, female gender, a body mass index (BMI) less than 23, a previous history of respiratory diseases such as tuberculosis or obstructive respiratory diseases (chronic obstructive pulmonary disease [COPD], asthma), and previous respiratory symptoms such as cough or dyspnea, were associated with high-risk lung radiology scores according to LDCT scans. These findings indicate that risk-based assessments using primary data and questionnaires to identify risk factors other than heavy smoking and a family history of lung cancer may improve the efficiency of lung cancer screening.

## 1. Introduction

In recent years, lung cancer has become the leading cause of cancer-related death in both men and women worldwide. The National Lung Screening Trial (NLST) demonstrated that annual low-dose computed tomography (LDCT) screening can reduce lung cancer mortality by 20% compared to chest radiography in heavy smokers with a history of at least 30 pack-years [1]. Following the NLST, the Taiwan Health Promotion Agency formulated a policy to subsidize LDCT lung cancer screening for individuals at a high risk of lung cancer, including heavy smokers and individuals with a family history of lung cancer. On 1 July 2022, the Ministry of Health and Welfare launched the Lung Cancer Early Detection Program to provide biannual low-dose computed tomography (LDCT) lung screening for high-risk groups. Taiwan is the first country to provide lung screening for heavy smokers and individuals with a family history of lung cancer. Those in the following groups at a high risk for lung cancer may apply for screening at any given hospital under the program: (1) Individuals with a family history of lung cancer, specifically, men aged between 50 and 74 years and women aged between 45 and 74 years whose parents, children, or siblings have been diagnosed as having lung cancer, and (2) individuals with a history of heavy smoking, specifically, individuals aged between 50 and 74 years with a smoking history of 30 or more pack-years who are willing to quit smoking or who have quit smoking within the past 15 years.

According to the National Lung Screening Trial (NLST) in the United States, it was found that individuals screened for lung cancer using low-dose CT had a 20% lower mortality rate compared to those screened using traditional X-rays. However, the study also found that 96.4% of the individuals with positive screening results had negative follow-up examinations. Therefore, developing a system for effectively managing and tracking positive screening results has become an important issue. In 2015, the American College of Radiology introduced a standardized, structured reporting system and corresponding management process for lung cancer screening, based on the successful concept of the Breast Imaging Reporting and Data System (BI-RADS) used in mammography screening. It is hoped that this system, called Lung-RADS, can effectively reduce the occurrence of false positive results.

While LDCT screening has been shown to be effective in reducing lung cancer mortality, not all individuals meet the criteria for LDCT screening as established by the Taiwan Health Promotion Agency. Accordingly, identifying risk factors for lung cancer is particularly important in individuals who may not be eligible for LDCT screening and may help identify individuals at high risk of developing lung cancer who may benefit from alternative screening strategies or preventative interventions.

The present study aimed to identify risk factors for lung cancer among individuals who do not meet the criteria for LDCT lung cancer screening in Taiwan. This may allow for the targeting of screening and preventative measures for individuals at a high risk of developing lung cancer.

## 2. Materials and Methods

This retrospective analysis included 12,542 asymptomatic healthy subjects who voluntarily underwent self-paid LDCT exams at the health check-up center of Kaohsiung Veterans General Hospital between January 2016 and December 2021. The study population comprised 6792 males and 4949 females aged 18–96 years, and excluded individuals who were heavy smokers or who had a family history of lung cancer.

Patients were classified into a high-risk group or low-risk group according to Lung-RADS score [2]. The high-risk group comprised cases with Lung-RADS scores of 3 and 4, with cases with a Lung-RADS score of 2 that had undergone follow-up examinations in thoracic medicine or thoracic surgery clinics within 6 months also assigned to the high-risk group. Cases with Lung-RADS scores of 1 and 2 who did not have follow-up examinations in thoracic medicine or thoracic surgery clinics within 6 months were assigned to the low-risk group.

Information, including gender, age, BMI, cigarette smoking habits, previous respiratory disease (including tuberculosis, asthma, and COPD), previous respiratory symptoms, cooking habits, and residential zone, was collected through a questionnaire. We then evaluated LDCT reports and clinical information from individuals in the high-risk and low-risk groups to verify whether the cases were diagnosed with lung cancer.

### Statistical Analysis

Baseline demographics and clinical characteristics were summarized for the entire analytical population (gender, age, BMI, cigarette smoking habits, previous respiratory disease, previous respiratory symptoms, cooking habits, and residential zone), divided into the high-risk group or not, and they are appropriately expressed as mean ± standard deviation or number (percentage). The differences between these two groups were compared using independent Student’s *t*-test for continuous and chi-squared test for categorical variables, respectively. In addition, significant determinants of the high-risk group were evaluated using univariate and multivariate logistic regression models. Multivariate logistic regression models were used to estimate adjusted odd ratios (ORs) with 95% confidence intervals (95% CIs).

All statistical analyses were performed using Statistical Analysis Software (SAS; version 9.4; SAS System for Windows) and SPSS (version 20; SPSS Inc., Chicago, IL, USA). A *p*-value < 0.05 was considered statistically significant.

## 3. Results

A summary of the baseline characteristics of the 12,542 individuals included in the present study is shown in Table 1. The high-risk group comprised 801 individuals, and the low-risk group comprised 11,741 individuals. The mean age of all the study participants was 53.0 ± 11.3 years, with a mean age of 56.7 ± 10.9 years in the high-risk group and 52.8 ± 11.2 years in the low-risk group. A statistically significant age difference was observed between the two groups (*p* < 0.001). A higher proportion of individuals in the high-risk group were over 55 years old (53%) compared to the low-risk group (42%; *p* < 0.001), with no significant difference in gender distribution observed between the two groups (*p* = 0.212). However, a higher proportion of individuals in the high-risk group were not overweight (BMI < 25, 68%) compared to individuals in the low-risk group (61%; *p* < 0.001). The proportion of individuals with previous chest symptoms (e.g., chest pain or tightness) did not significantly differ between the two groups (*p* = 0.218). Statistically significant differences in the prevalence of previous respiratory symptoms such as coughing (*p* = 0.001) and dyspnea or breathlessness when exercising (*p* = 0.001) were observed between the two groups. A higher proportion of individuals in the high-risk group reported previous respiratory symptoms than those in the low-risk group. A higher proportion of individuals in the high-risk group had underlying respiratory disease (e.g., asthma, tuberculosis, or obstructive pulmonary disease) compared to the low-risk group (*p* < 0.001). No significant difference in smoking habits was observed between the two groups (*p* = 0.218). However, a higher proportion of individuals in the high-risk group reported a high-risk cooking habit than the low-risk group (*p* < 0.001). No significant difference in residential location was observed between the two groups (*p* = 0.286).

The results of the logistic regression analysis are presented in Table 2. This logistic regression model identified several variables significantly associated with high-risk Lung-RADS scores in univariate and multivariate models. In the univariate model, age, sex, BMI, previous respiratory symptoms, underlying respiratory disease, and cooking habits were all significant predictors of high-risk Lung-RADS scores. Individuals over the age of 55 years had 1.61-fold higher odds of having high-risk Lung-RADS scores compared to individuals aged 55 years or younger. Females had 1.38-fold higher odds of having high-risk Lung-RADS scores compared to males. Similarly, individuals who were not overweight (BMI < 25) had 1.36-fold higher odds of having high-risk Lung-RADS scores compared to individuals who were overweight (BMI ≥ 25). Individuals with previous chest pain or tightness had 1.35-fold higher odds of having high-risk Lung-RADS scores than those without. Individuals with COPD or pulmonary tuberculosis were more likely to have high-risk Lung-RADS scores than individuals with COPD or pulmonary tuberculosis. Finally, individuals who engaged in high-risk cooking practices had 1.47-fold higher odds of having high-risk Lung-RADS scores than those who engaged in low-risk cooking practices.

In the multivariate model, age, sex, BMI, and previous respiratory symptoms remained significant predictors of high-risk Lung-RADS scores. Individuals over the age of 55 years had 1.56-fold higher odds of having high-risk Lung-RADS scores compared to individuals who were aged 55 years or younger. Females had 1.30-fold higher odds of having high-risk Lung-RADS scores compared to males. Individuals who were underweight (BMI < 25) had 1.29-fold higher odds of having high-risk Lung-RADS scores compared to those who were overweight (BMI ≥ 25). Individuals with chest pain or tightness had 1.25-fold higher odds of having high-risk Lung-RADS scores than those without. These results indicate that age, sex, BMI, and previous respiratory symptoms are associated with high-risk Lung-RADS scores according to LDCT scans.

**Figure 1:**
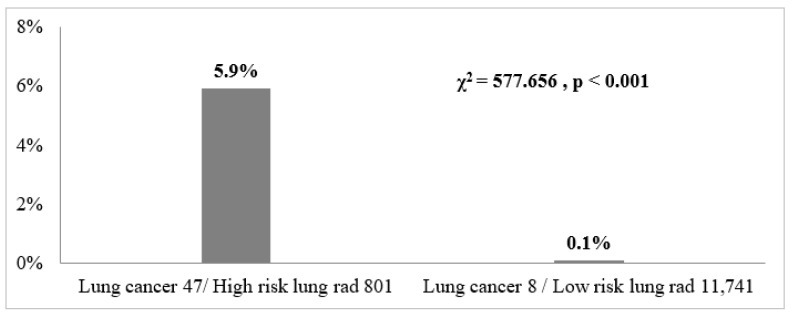
The density histogram depicts all individuals divided into two groups: one categorized as “high-risk of lung cancer by CT radiography” and the other as “low-risk of lung cancer by CT radiography.” The histogram further records the proportions of cases within each group that have developed lung cancer.

Of the 12,542 individuals, 55 had lung cancer, with 47 cases detected in the high-risk group and 8 in the low-risk group (Figure 1). The diagnosis of lung cancer was made through advanced biopsy or thoracic surgery within the last year. A statistically significant difference in the prevalence of lung cancer was observed between the two groups (Pearson’s chi^2^ = 577.6555, Pr < 0.0001). These findings indicate that individuals in the high-risk group based on radiographic abnormalities on LDCT may be more likely to develop lung cancer.

**Figure 2:**
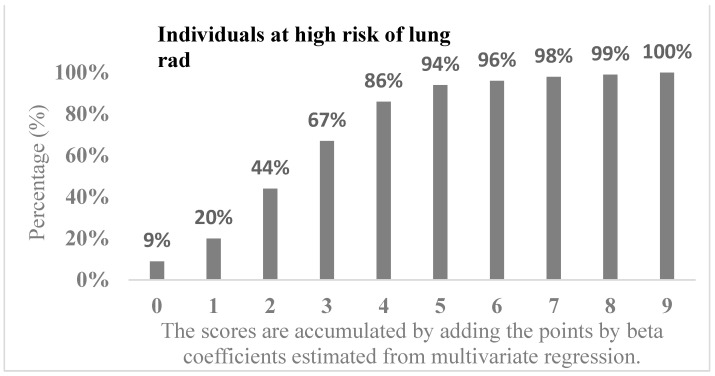
The density histogram describes how the scores are accumulated by adding points based on beta coefficients estimated from multivariate regression. The histogram further records the cumulative percentage of cases related to a high risk of lung cancer by CT radiography.

The following factors were found to be associated with an increased Lung-RADS score (presented in Table 3): age over 55 years (2 points), female gender (1 point), not overweight BMI (1 point), previous respiratory symptoms (1 point), presence of obstructive pulmonary disease (2 points), and presence of pulmonary tuberculosis (4 points). The number of points assigned to each factor was determined by beta coefficients from the multivariate regression analysis. Individuals who are older, female, underweight, and have significant respiratory symptoms or underlying respiratory disease had higher scores, indicating a higher likelihood of developing lung cancer. The presence of pulmonary tuberculosis was found to have the largest impact on the risk of lung cancer, with 4 points. The high-risk group comprised 86% and 94% of the individuals who scored more than 4 and 5 points, respectively. This result indicates individuals with a score of 5 or more according to questionnaire answers and basic information should undergo LDCT, as a high-risk Lung-RADS score is more likely (Figure 2).

## 4. Discussion

In 2013, the United States Preventive Services Task Force recommended annual lung cancer screening with LDCT for smokers aged between 55 years and 80 years with at least 30 pack-years of smoking exposure who currently smoke or have quit smoking within the previous 15 years [3].

Age is closely associated with the incidence of lung cancer, with older individuals having higher rates of lung cancer than younger individuals. Further, histologic subtypes of lung cancer have differing age distributions [4]. The incidence and mortality of lung cancer increase with age, with the highest rates observed among individuals in their eighth and ninth decades of life. This is likely due to a combination of factors, including longer exposure to tobacco smoke and other environmental factors that increase the risk of lung cancer [5]. Overall, the results of these studies indicate that age is an important factor in the development and prognosis of lung cancer and that early detection and intervention may improve outcomes, particularly among older individuals at a higher risk of developing lung cancer.

Previous studies have suggested that women who smoke are at a higher risk of developing lung cancer than men who smoke, while other studies have found no significant difference between genders. Studies conducted in 1993 and 1994 by Risch et al. found that women who smoke are at a higher risk of developing lung cancer than men who smoke, particularly for certain subtypes of lung cancer [6,7]. A study by Bain et al. in 2004 found that women have a higher risk of developing lung cancer than men with similar smoking histories, indicating that females may be more susceptible to lung cancer than males [8]. A further study found that female smokers are more likely to develop lung cancer than male smokers, suggesting that the increased risk may be related to gender-specific differences in biological or hormonal factors [9]. Zang and Wynder also found that women have a higher risk of developing lung cancer than men and suggested that this difference may be due to variations in smoking habits, hormonal factors, or other biological differences [10]. Taken together, these studies indicate that females who smoke are at a higher risk of developing lung cancer than male smokers and that biological or hormonal factors may contribute to this difference. However, more research is required to fully understand the reasons for the higher risk of developing lung cancer in females and to develop effective prevention strategies. A 2013 study by De Matteis et al. found no significant difference in the risk of developing lung cancer between male and female smokers after adjusting for smoking intensity and duration [11]. While there may be some evidence suggesting that women who smoke are at a higher risk of developing lung cancer than men who smoke, this evidence is inconsistent across studies.

A cohort study from China and the United States that used data from the UK Biobank to examine the associations of genetic risk, BMI trajectories, and the risk of non-small cell lung cancer (NSCLC) found that both genetic risk and BMI trajectories were independently associated with NSCLC risk and that the joint effects of genetic risk and BMI trajectories were stronger than their individual effects. They also found that BMI trajectories modified the effects of genetic risk on NSCLC risk and that the highest risk was observed among individuals with high genetic risk and increasing BMI trajectories [12].

A large-scale randomized controlled trial based on data from the National Lung Screening Trial (NLST) that evaluated the effectiveness of low-dose CT screening for lung cancer reported a difference in the association between BMI and lung cancer diagnosis according to ethnicity. Specifically, a higher BMI was associated with a lower risk of lung cancer diagnosis among non-Hispanic white participants but not among Black participants. The study also found that Black participants had a higher risk of lung cancer diagnosis than non-Hispanic white participants, even after adjusting for BMI and other factors [13].

A separate study used data from the HUNT study to examine the causal association between BMI and lung cancer incidence using observational and Mendelian randomization approaches and found that BMI was inversely associated with lung adenocarcinoma but not with other lung cancer types [14].

Previous studies have reported a strong association between COPD and the risk of developing lung cancer, with our results corroborating these previous findings. For example, a study of a nationally representative cohort of the US population with up to 22 years of follow-up found that moderate or severe obstructive lung disease was associated with an increased risk of incident lung cancer [15]. The coexistence of chronic obstructive pulmonary disease (COPD) and lung cancer is characterized by shared risk factors, including tobacco smoke exposure and genetic predispositions. Chronic inflammation, oxidative stress, epigenetic alterations, dysregulated cell signaling, and altered immune responses contribute to their comorbidity. Understanding these molecular mechanisms is crucial for developing effective therapeutic strategies and improving clinical outcomes [16]. The presence of comorbidities such as tuberculosis in COPD patients may also be related to the increased risk of lung cancer. It has been suggested that COPD patients with a history of tuberculosis, particularly never-smokers, may benefit from regular screening or evaluation for the development of lung cancer [17].

Participants with tuberculosis (TB) sequelae had a higher number of nodules and higher emphysema rates than those without TB sequelae. Additionally, the proportion of individuals with positive screening results was higher among participants with TB sequelae. The authors concluded that TB within a particular population should be considered when interpreting lung cancer screening results [18].

A 2015 study by Kocher et al. investigated the presenting symptoms of patients with NSCLC and found that cough was present in 50–75% of patients with lung cancer and that a cough productive of large volumes of thin, mucoid secretions was seen in patients with mucinous adenocarcinoma [19].

Ying-Chin Ko et al. found that exposure to Chinese food cooking fumes was associated with an increased risk of lung cancer in nonsmoking women, highlighting the potential health risks associated with traditional cooking methods in Chinese cuisine [20]. Yingbo Xue et al. conducted a meta-analysis of eight studies and confirmed a significant association between cooking oil fume exposure and lung cancer risk in Chinese nonsmoking women [21]. The authors posited that this association may be due to the production of carcinogenic substances, such as PAHs, during high-temperature cooking. Yu et al. also found a dose–response relationship between cooking fume exposure and lung cancer risk in Chinese nonsmoking women, further supporting the potential health risks associated with cooking [22]. The results of these studies highlight the importance of understanding the potential health risks associated with cooking, particularly for Chinese nonsmoking women who may be at increased risk of developing lung cancer due to traditional cooking methods [23]. However, Bigert et al. found no statistically significant increase in the overall risk of developing lung cancer among individuals who regularly cook when accounting for smoking in men or women, with no association between exposure duration and risk of lung cancer [24].

Previous studies have indicated strong evidence linking outdoor air pollution, particularly PM 2.5 and NOx, with an increased risk of various types of cancer, including lung, bladder, and cardiovascular disease [25,26,27]. One study reported that there was no difference in the incidence of lung cancer between different regions of Taiwan. However, this study focused on the relationship between air pollution and lung cancer in nonsmokers throughout Taiwan rather than regional variations [28].

Owing to the retrospective nature of this study, it is imperative to acknowledge certain limitations inherent in it. The primary limitation lies in the reliance on the 2014 version of LUNG RAD (Radiology) for the interpretation and judgment of findings within the reports. Despite the availability of more recent versions, this study adheres to the standards and criteria established by healthcare professionals during the retrospective period. Consequently, the exclusion of newer versions of LUNG RAD may limit the generalizability of findings to the latest diagnostic advancements. This limitation underscores the importance of considering the evolving nature of medical standards and the potential impact on the interpretation of results.

Lung cancer is a major global health concern, and lung cancer screening effectively reduces mortality in high-risk populations. However, not all individuals may meet the criteria for LDCT screening as established by the Taiwan Health Promotion Agency. The present study identified potential risk factors for lung cancer among individuals who do not meet these criteria, including age, gender, and previous medical history. By considering these additional risk factors and symptoms related to the previous respiratory system, it may be possible to identify individuals at a high risk of developing lung cancer who may benefit from earlier diagnosis and treatment.

## 5. Conclusions

Although LDCT screening has been shown to be effective in improving lung cancer outcomes, further research is required to determine optimal screening intervals and protocols for specific populations. Long-term follow-up using LDCT may also provide valuable information regarding changes in lung health over time and the incidence of lung cancer in high-risk populations. By continuing to identify novel risk factors for lung cancer and refining screening strategies, it may be possible to further reduce the burden of lung cancer and improve patient outcomes.

## Figures and Tables

**Table 1:** Characteristics at baseline.

	Total	With High-Risk Group	Without High-Risk Group	
Variables	N = 12,542 (%)	N = 801 (%)	N = 11,741 (%)	*p*-Value
Age (mean ± SD), year	53.0 ± 11.3	56.7 ± 10.9	52.8 ± 11.2	<0.001
Age				<0.001
≤55	7234 (58)	374 (47)	6860 (58)	
>55	5308 (42)	427 (53)	4881 (42)	
Sex				<0.001
Male	7192 (57)	400 (50)	6792 (58)	
Female	5350 (43)	401 (50)	4949 (42)	
BMI				<0.001
Not overweight (<25)	7722 (62)	546 (68)	7176 (61)	
Overweight (25–29)	4820 (38)	255 (32)	4565 (39)	
Previous chest symptom *				0.212
Yes	2389 (19)	166 (21)	2223 (19)	
No	10,153 (81)	635 (79)	9518 (81)	
Previous respiratory symptom				0.001
Cough	2303 (18)	176 (22)	2127 (18)	
Dyspnea/breathless when exercising	1399 (11)	108 (14)	1291 (11)	
No	8840 (71)	517 (65)	8323 (71)	
Underlying chest disease				<0.001
Asthma	409 (3)	40 (5)	369 (3)	
Tuberculosis	157 (1)	27 (3)	130 (1)	
Obstructive pulmonary disease	204 (2)	18 (2)	186 (2)	
No	11,772 (94)	716 (89)	11,056 (94)	
Smoking habit				0.218
≥20 pack-year	10,532 (84)	685 (86)	9847 (84)	
No smoking Or <20 pack-year	2010 (16)	116 (15)	1894 (16)	
Residential location				0.286
Living in South Taiwan	9288 (74)	606 (76)	8682 (74)	
Not living in South Taiwan	3254 (26)	195 (24)	3059 (26)	
Cooking habit				<0.001
High-risk	1794 (14)	154 (19)	1640 (14)	
Low-risk	10,748 (86)	647 (81)	10,101 (86)	

* Significant chest symptom includes chest pain or chest tightness.

**Table 2:** Odds of being in high-risk group by RADS.

	Univariate		Multivariate	
Variables	OR (95% CI)	*p*-Value	aOR (95% CI)	*p*-Value
Age				
≤55	Ref		Ref	
>55	1.61 (1.39–1.85)	<0.001	1.56 (1.35–1.80)	<0.001
Sex				
Male	Ref		Ref	
Female	1.38 (1.19–1.59)	<0.001	1.30 (1.12–1.51)	0.001
BMI				
Not overweight (<25)	1.36 (1.17–1.59)	<0.001	1.29 (1.10–1.51)	0.002
Overweight (≥25)	Ref		Ref	
Previous respiratory symptom				
Yes	1.35 (1.16–1.56)	<0.001	1.25 (1.08–1.46)	0.004
No	Ref		Ref	
Underlying chest disease				
Obstructive pulmonary disease	1.54 (1.16–2.03)	0.003	1.48 (1.11–1.97)	0.007
Pulmonary tuberculosis	3.10 (2.03–4.70)	<0.001	2.72 (1.78–4.16)	<0.001
No	Ref		Ref	
Smoking habit				
No smoking Or <20 pack-year	Ref			
≥20 pack-year	0.88 (0.72–1.08)	0.218		
Residential location				
Living in South Taiwan	1.10 (0.93–1.29)	0.286		
Not living in South Taiwan	Ref			
Cooking habit				
High-risk	1.47 (1.22–1.76)	<0.001		
Low-risk	Ref			

**Table 3:** Points are estimated by beta coefficients from multivariate regression.

Variables	Points
Age > 55	2
Female	1
Underweight (<25)	1
Previous respiratory symptom	1
Obstructive pulmonary disease	2
Pulmonary tuberculosis	4

## Data Availability

All results are available from the Kaohsiung Veterans General Hospital. The database used for the study can be made available from the corresponding author under request if needed.
